# Genome Sequences of Two *Pseudomonas syringae* pv. tomato Race 1 Strains, Isolated from Tomato Fields in California

**DOI:** 10.1128/genomeA.01671-15

**Published:** 2016-03-10

**Authors:** Shree P. Thapa, Gitta Coaker

**Affiliations:** Department of Plant Pathology, University of California Davis, Davis, California, USA

## Abstract

*Pseudomonas syringae* pv. tomato race 1 strains have evolved to overcome genetic resistance in tomato. Here, we present the draft genome sequences of two race 1 *P. syringae* pv. tomato strains, A9 and 407, isolated from diseased tomato plants in California.

## GENOME ANNOUNCEMENT

*Pseudomonas syringae* pv. tomato causes bacterial speck of tomato, which is one of the most persistent bacterial diseases in tomato worldwide. In resistant genotypes, the race 0 *P. syringae* pv. tomato type III effectors AvrPto and AvrPtoB are recognized by the tomato proteins Pto and Prf (1, 2). However, *P. syringae* pv. tomato race 1 strains are able to overcome genetic resistance in tomato by modifying the presence and expression of AvrPto and AvrPtoB (3, 4). Despite the historical success of Pto-mediated resistance, race 1 strains now predominate (4, 5). *P. syringae* pv. tomato race 1 was first detected in 1986 in Canada and in 1993 in California, the primary production area for processing tomato cultivars in the United States (6, 7). The vast majority of strains collected from 2005 to 2007 were race 1, and we were unable to identify any race 0 strains in 2007, 2008, or 2009 from infected tomato plants in California (4, 5). Here, we report the draft genome sequences of *P. syringae* pv. tomato A9 and *P. syringae* pv. tomato 407, isolated from infected tomato plants in California. Both strains are race 1, but *P. syringae* pv. tomato A9 exhibits enhanced bacterial growth and disease symptoms in tomato compared to *P. syringae* pv. tomato 407 (8).

Genomic DNA was sequenced using the Illumina HiSeq 2500 (2 × 150-bp paired-end reads) at the Genome Center at the UC Davis DNA Technologies Core Facility. After the raw sequences were trimmed and their quality filtered (>Q30), the remaining reads were assembled *de novo* using the SPAdes assembler and draft genomes were generated for each isolate (9). Each genome was annotated with PROKKA (10) and the NCBI Prokaryotic Genome Annotation Pipeline (PGAP) (http://www.ncbi.nlm.nih.gov/genome/annotation_prok).

The final draft assembly of the *P. syringae* pv. tomato A9 genome consists of 188 contigs (>200 bp) with 70-fold genome coverage. *P. syringae* pv. tomato A9 harbors a single circular genome of 6,314,445 bp, with a G+C content of 57.9%. The genome of the *P. syringae* pv. tomato A9 strain contains 5,749 predicted coding sequences (CDSs), 1 rRNA operon, and 60 tRNA genes; and the genome of *P. syringae* pv. tomato 407 contains 5,702 predicted CDSs, 1 rRNA operon, and 57 tRNA genes. The final draft assembly of the *P. syringae* pv. tomato 407 genome consists of 192 contigs (>200 bp) with 65-fold genome coverage. *P. syringae* pv. tomato 407 harbors a single circular genome of 6,264,873 bp with a G+C content of 55.8%. Among the 57 type III effectors present in the *P. syringae* pangenome (11), 27 are present in both *P. syringae* pv. tomato A9 and *P. syringae* pv. tomato 407. Detailed comparisons of related *Pseudomonas* strains exhibiting variable virulence will facilitate insight into molecular mechanisms regulating virulence and adaptation.

### Nucleotide sequence accession numbers.

The sequences have been deposited as whole-genome shotgun projects in GenBank under the accession numbers LNKY00000000 for *P. syringae* pv. tomato A9 and LNKZ00000000 for *P. syringae* pv. tomato 407.
